# Clinical and Biological Interpretation of Survival Curves of Cancer Patients, Exemplified With Stage IV Non-Small Cell Lung Cancers With Long Follow-up

**DOI:** 10.3389/fonc.2022.837419

**Published:** 2022-02-02

**Authors:** Jan P. A. Baak, Hegen Li, Huiru Guo

**Affiliations:** ^1^ Department of Pathology, Stavanger University Hospital, Stavanger, Norway; ^2^ Medical Practice Dr. Med Jan Baak AS, Tananger, Norway; ^3^ Department of Medical Oncology, Longhua University Hospital, Shanghai, China

**Keywords:** metastatic cancer, non-small cell lung cancer, stage IV, survival curve analysis, detection of different subgroups

## Abstract

Worldwide, 18.1 million new invasive cancers and 9.9 million cancer deaths occurred in 2020. Lung cancer is the second most frequent (11.4%) and, with 1.8 million deaths, remains the leading cause of cancer mortality. About 1.7 million of lung cancers are of the non-small cell lung cancer (NSCLC) subtype, and of these, 60%–70% are in advanced stage IV at the time of diagnosis. Thus, the annual worldwide number of new NSCLC stage IV patients is about 1 million, and they have a very poor prognosis. Indeed, 25%–30% die within 3 months of diagnosis. However, the survival duration of the remaining 700,000 new patients per year surviving >3 months varies enormously. Surprisingly, little research has been done to explain these survival differences, but recently it was found that classical patient, tumour and treatment features cannot accurately distinguish short- and very long-term survivors. What then are the causes of these bewildering survival variations amongst “the same cancers”? Clonality, proliferation differences, neovascularization, intra-tumour heterogeneity, genetic inhomogeneity and other cancer hallmarks play important roles. Considering each of these, single or combined, can greatly improve our understanding. Another technique is analysis of the survival curve of a seemingly homogeneous group of cancer patients. This can give valuable information about the existence of subgroups and their biological characteristics. Different basic survival curves and what their shapes tell about the biological properties of these invasive cancers are discussed. Application of this analysis technique to the survival curve of 690 stage IV NSCLC patients with a 3.2–120.0-month survival suggests that this seemingly homogeneously group of patients probably consists of 4–8 subgroups with a very different survival. A subsequent detailed mathematical analysis shows that a model of 8 subgroups gives a very good match with the original survival curve of the whole group. In conclusion, the survival curve of a seemingly homogeneous group of cancer patients can give valuable information about the existence of subgroups and their biological characteristics. Application of this technique to 690 NSCLC Stage IV patients makes it probable that 8 different subgroups with very different survival rates exist in this group of cancers.

## Introduction

Worldwide, an estimated 18.1 million new invasive cancer cases and almost 9.9 million cancer deaths occurred in 2020. The average mortality rate of invasive cancers is therefore about 50%. Lung cancer, the second most frequently occurring cancer at 11.4%, with an estimated 1.8 million deaths, remains by far the leading cause of cancer mortality (1). Similar rates for lung cancer are found in the People’s Republic of China (2). About 1.7 million of the lung cancers are of the non-small cell lung cancer (NSCLC) subtype, and of these, 60%–70% are in advanced stage IV at the time of diagnosis. Thus, the annual worldwide number of new NSCLC stage IV patients is about 1 million and they are generally regarded as having a very poor prognosis. Indeed, 25%–30% die within 3 months of diagnosis. On the other hand, the survival duration of those remaining approximately 700,000 new patients per year surviving >3 months can vary enormously. In a recent large observational study, median survival was 23.3 months, 1-, 2- and 5-year survival rates are 74%, 49% and 16% respectively and 4%–5% survive 10 years and longer (3). The same surprising enormous survival variation can be found in patients with cancers from other organ sites, even if they have the same histological type, stage and other important prognostic characteristics.

What are the causes of these bewildering survival variations amongst “the same cancers”?

An important aspect is *clonality*. Cancer is an evolutionary process, driven by stepwise, somatic cell mutations with sequential, sub-clonal selection (4). Normal, polyclonal cells have approximately the same proliferation rate. However, sometimes genetic hits occur and change the polyclonal parent cell into neoplastic daughter cells with a new genetic make-up plus growth (proliferation) advantage. As a result, a very small nodule arises, consisting of cells which are genetically somewhat more unstable. Consequently, the risk of the development of another new cell clone with even more genetic instability and higher proliferation, and eventually invasive capacity, increases. These new tumour cells grow in densely packed populations that develop into spheroid or ellipsoid aggregates.

Another important aspect is *neovascularization*. This is an event that separates the development of any solid tumour into two stages: the avascular stage and the vascular stage. Because of this, angiogenesis plays a critical role in the biology of solid neoplasms. The two stages can be dissociated under experimental conditions. When this is accomplished and capillaries are prevented from penetrating the l-mm tumour, the tumour becomes dormant (5, 6).

This led to the concept of dormant cancers (7). Dormant solid tumours were produced *in vivo* by prevention of neovascularization. The beginning of an exponential volume increase was shown to coincide with vascularization of the implant. Although dormant in terms of expansion, these avascular tumours contained a population of viable and mitotically active tumour cells.

The transition from polyclonal to neoplastic cells probably occurs quite often. How long it takes to change from a 1mm diameter dormant tumour (consisting of approximately 1 million cancer cells) to a clinically detectable proliferating invasive cancer, of approximately 10 (7.5–15.0) mm (10^9^ tumour cells), is less certain. From there to lethal metastases of 1,000 g (rough estimate), or 10^12^ cells, depends amongst other factors on *intratumour heterogeneity (ITH)* (8). *Genomic diversity* within single tumours has been recognized as “*genetic inhomogeneity*” (9). Since next-generation sequencing studies have become available, the full extent of genomic ITH is becoming apparent. The degree of ITH can be highly variable, with between 0 and over 8,000 coding mutations found to be heterogeneous within primary tumours or between primary and metastatic or recurrence sites (10). These findings make it more than likely that especially seemingly homogeneous late-stage cancers are, in fact, genetically widely heterogeneous, also in their clinical behaviour. The latter can be observed in the survival curve of these cancers.

It is of obvious clinical and therapeutic significance to understand why patients, with seemingly homogeneous cancers, have such different survival rates. Of course, often, age, gender, performance stage, histologic subtype, no/minimal versus heavy smoking and different treatment modalities are strongly prognostic. In pulmonary adenocarcinoma, the mean number of clonal and sub-clonal non-silent mutations in non-smokers is much smaller than in smokers (8). However, even when these well-established prognostic factors are all considered, also in a multivariate manner, it may not be possible to explain why certain patients die within a rather short time, while others survive for (very) many years, as we recently found. It is important to emphasize that the number of these patients worldwide is very large indeed.

We recently worked on an article on the survival prediction accuracy of prognostic factors in the seemingly homogeneous group of 690 stage IV NSCLC surviving patients between 3.2 and 120.0 months. In the original manuscript, we hypothesized that this group in fact consisted of several hypothetical subgroups with widely varying survival rates. This hypothesis was based on the interpretation of the survival curve of the patients (3).

Survival curves can give valuable information about the clinical behaviour and the biological characteristics of a group of cancer patients. Such biological interpretation of survival curves was common knowledge in the last 2–3 decades of the 20th century. In fact, the first author of the current manuscript taught this knowledge as a standard part of the curriculum for medical students in Amsterdam. However, the comments of the reviewers of our recent manuscript (3), on our remarks to identify different prognostic subgroups by analysis of the survival curve, made it clear that this survival curve analysis knowledge was not as well-known as we thought. Rather than writing a long new section in that article to explain how we had come to the hypothesis of the existence of 4–8 subgroups with different survival rates, it was advised by the Acting Editor of the revised version of the manuscript that the topic of *Clinico-biologic Interpretation of Survival Curves* would be interesting enough for a separate new manuscript.

This article will first describe different types of survival curves and how to analyse and classify them using essential hallmarks of cancer. Secondly, we will perform quantitative model studies to show that in seemingly homogeneous stage IV NSCLC patients with a 3.2–120.0-month follow-up, about 4–8 subgroups with very different survival rates occur.

## Different Types of Survival Curves and Their Clinico-Biologic Interpretation

We will only consider tumours diagnosed as invasive carcinomas. Please remember that although the examples are hypothetical, many “real-world” examples can be found for each of them.

**Figure 1:**
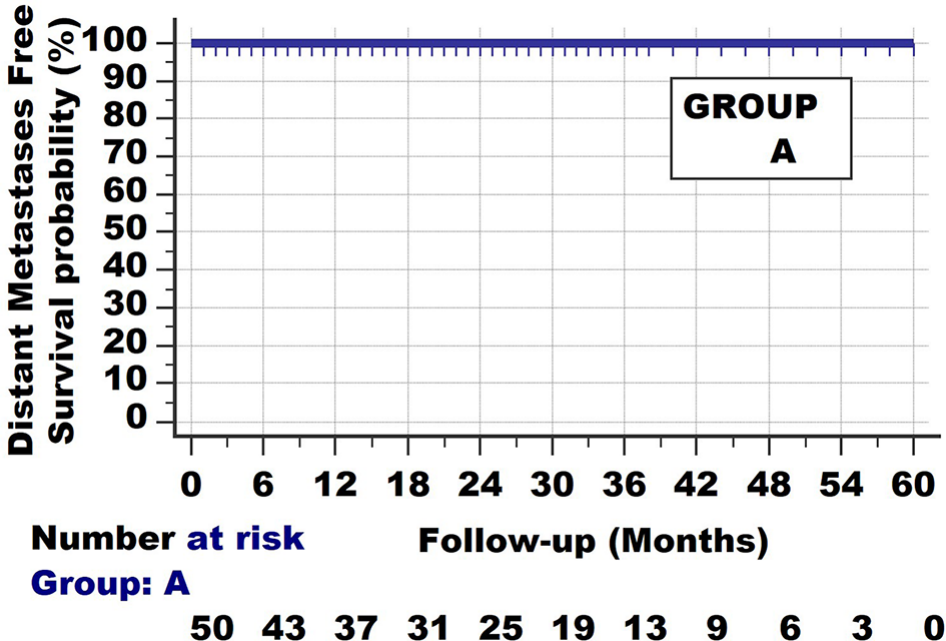
Invasive cancer group A. Survival curve with long follow-up. None of the patients develop/die from metastatic disease.

Basically 2 fundamental types of survival curves exist. The first one is shown in **Figure 1**. The cancer can be detected by the patients when the tumour reaches a certain size, or by radiologic and other screening methods. When the tumours are removed, histopathologic examination will show the invasive nature of the tumour. In the following years, none of the patients develop distant metastases; all survive without evident distant metastases.

**Figure 2:**
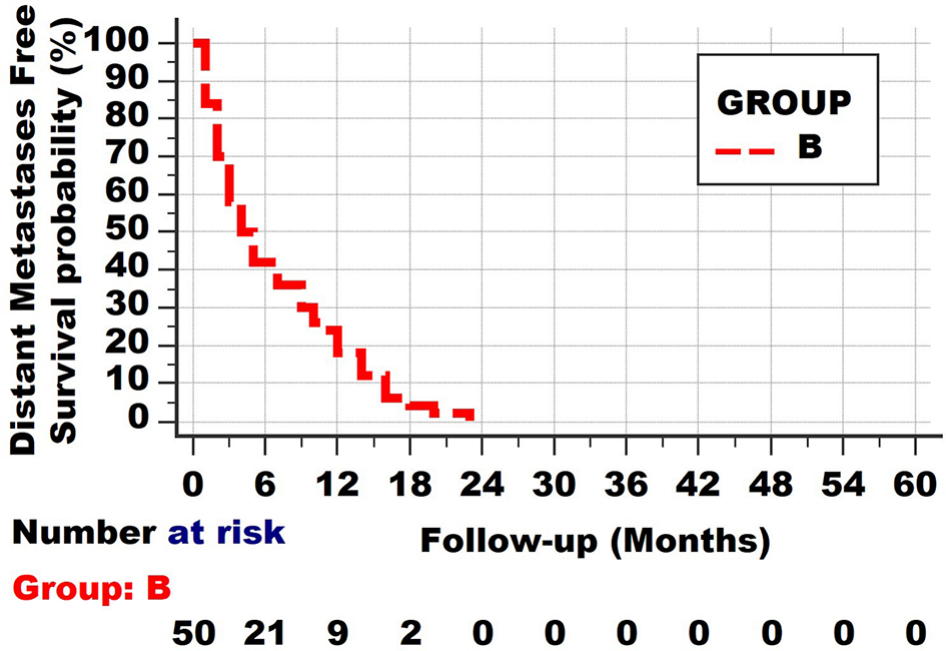
Invasive cancer group B All patients have died from metastatic disease within 24 months.

The second extreme example of a survival curve type is of patients with cancers, shown in **Figure 2**. One can think of small cell lung cancer. All have died from their metastases at the end of the observation period (which in the current hypothetical example was set at 23 months but can also be set at 6 or 12 months). They seem homogeneous at the start of the follow-up, yet they have considerable differences in survival rates. 60% have died by the 6-month follow-up, 20% between 6 and 12 months and the last 20% between 12 and 23 months.

**Figure 3:**
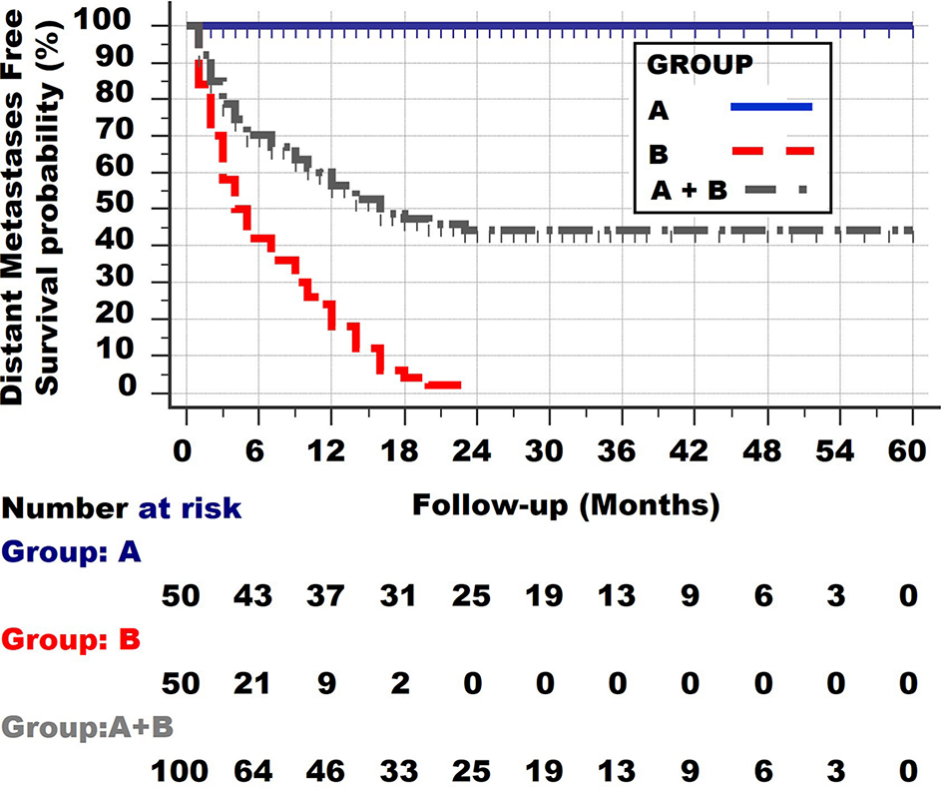
Survival curve of the two patient groups A and B together. Note the initial rather steep decline, followed by an increasingly more horizontal plateau.

**Figure 3** shows the third type of survival curve, which occurs quite often. 30% of this group dies within 6 months, 10% between 6 and 12 months and 12% between 12 and 23 months. The remaining 45% of the patients survive until the end of the observation period. Such a curve is found when patients from the 2 different groups A and B are taken together.

It can be concluded from the shape of the survival curves shown in **Figure 3** that a group with a curve with an initial steep decline, followed by a horizontal plateau, consists of one subgroup with a long distant metastases survival and 3 other groups with a very poor, poor and less poor survival.

The most basic biologic interpretation of Group A and B is that they both show *Invasion*, as they are pathologically diagnosed as invasive cancers.

They also all have *clonal expansion.* Clonal expansion is not limited to invasive cancers but also occurs in non-invasive neoplasias, such as for endometrial intraepithelial neoplasia (11).

Cancers from Group B patients not only have invasive and clonal expansion properties, just like those from group A, but also all have *distant metastases* at the time of the diagnosis. That the net growth of these metastases differs in these group B cancers is clear from the shape of the survival curve, as most of the patients die from their metastases once these have reached a certain lethal level (which is on average 1–2 kg, although much greater weights can be found in individual patients). (Some patients will die from much smaller tumours if they are located at vitally essential locations, but these are exceptions). The 60% deaths in the first 6 months have on average reached their lethal metastatic mass within 6 months. Of course, the original volume at diagnosis may have varied, but the most important feature of these 60% of the tumours, compared with the other 20% dying between 6 and 12 months, is their higher net growth (the balance between the proliferation rates and death rates of the tumour cells). Likewise, the patients dying between 12 and 23 months again have a lower net growth rate. One can thus conclude that Group B tumours are both invasive, clonally expanding but also metastatic. One can further conclude from the survival curve of group B that it is not completely homogeneous but still consists of at least 3 subgroups with different proliferation rates (net growth speeds): very fast, fast and less fast.

**Figure 4:**
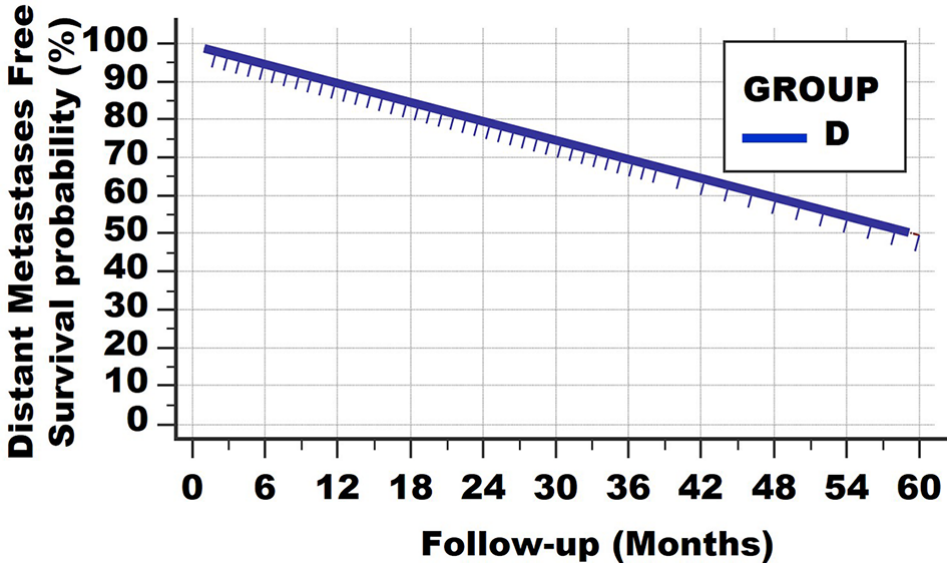
Survival curve type 4. At no point is a horizontal plateau found. This means that all patients had (occult) metastases at the time of diagnosis. However, the growth speeds vary greatly, resulting in continuous deaths from lethal metastatic load.

A fourth type of survival curve is shown in **Figure 4** of a hypothetical group D. At no point is a horizontal plateau found in the survival curve. Instead, at the end of the observation period 50% have died from distant metastases. On the other hand, the slope of the survival curve is much less steep than in the first, second and third subgroups of Group B. The conclusion is that patients of this group D all have distant metastases at the time of diagnosis, but with much lower net growth speeds than those of the subgroups of Group B. Alternatively, one could argue that the metastatic load of patients from Group B was much larger at the time of diagnosis. These 2 features cannot be discerned with the survival curves.

Of course, such a linear curve can be found with different follow-up times, for example 10, 20 and 30 years. Examples are Hodgkin-type lymphomas and certain breast cancers.

## Application of Survival Curve Analysis in Stage IV NSCLC

As described before, about 1.7 million of the lung cancers are NSCLC, and of these, 60%–70% are in advanced stage IV at the time of diagnosis. Thus, the annual worldwide number of new NSCLC stage IV patients is close to 1 million (3). 25%–30% die at <3 months. Yet, of those annually worldwide 700,000 NSCLC stage IV surviving >3 months, 10%–15% (70,000–105,000 new patients worldwide per year) survive >5 years. Surprisingly, little scientific attention has been paid to the question: which factors cause the good prognosis in these NSCLC stage IV-long survivors? In a non-interventional study of 998 consecutive first-onset stage IV NSCLC patients, a large group of 737 stage IV NSCLC patients with very long follow-up (survivals were 3.2–120.0 months), we investigated the accuracies of short- and long-term survival predictive values of baseline factors, radiotherapy (RT), platinum-based chemotherapy (PBT) and tyrosine kinase inhibitors targeted therapy (TKI-TT). Of the 737 patients surviving 3.2–120.0 months, 47 refused radiotherapy, platinum-based therapy and tyrosine kinase inhibitor-targeted therapy (TKI-TT). The median survival (16.1 months) of the 47 patients who refused PBT, RT and TKI-TT was significantly worse than of those with RT, PBT and/or TKI-TT (23.3 months, HR = 1.60, 95% CI = 1.06–2.42, *p* = 0.04). Of these latter 690 patients, 42% were females, 58% males, median age 63 (range 27–85) years, 1-, 2-, 5- and 10-year survival rates 74%, 49%, 16% and 5%, respectively. 16% were alive with disease (AWD) at the last follow-up. Pathology subtype (adenocarcinoma vs. all others), performance score, TNM substage, the number of PBT cycles and TKI-TT had independent predictive value. However, with the multivariate combination of these features, identification results of short-term non-survivors and long-term survivors were poor.

**Figure 5:**
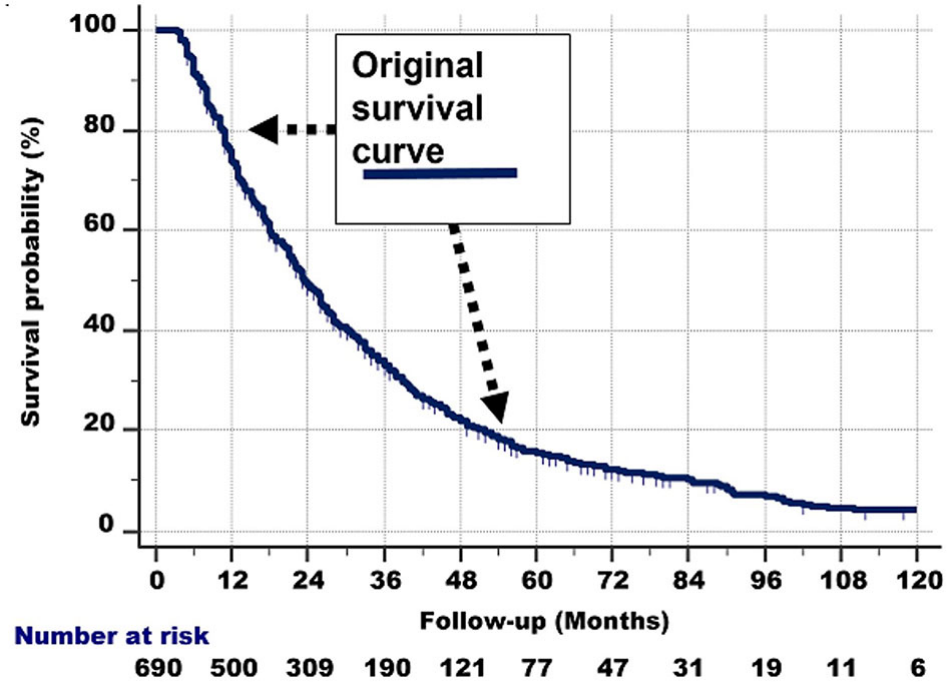
*Left:* Survival curve of the whole group of 690 stage IV NSCLC patients with a 3.2–120.0-month follow-up, who had received conventional radiotherapy, platinum-based chemotherapy and tyrosine kinase inhibitor-targeted therapy.

The shape of the survival graph of the 690 patients (**Figure 5**, left part) is curved. As described above, this suggests that the seemingly homogeneous group of 690 patients in fact is heterogeneous, i.e., is comprised of different subgroups with widely different survivals.

A closer inspection of **Figure 5**, left part, shows the following:

1. The survival line is almost straight and decreases steeply from 100% survival probability at the 3-month follow-up, to 70% at 12 months.
2. From that point, the survival curve still goes down, but less steeply. This second nearly straight line is between 70% at 12 months and 45% survival probability at the 30-month follow-up.
3. Then, after another bend, the curve is nearly straight between 45% and 28% survival probability (the latter is at about 45 months of follow-up). The slope of this third line again is less steep.
4. Between 28% and 18% survival probability (follow-up at 45% and around 55–60 months), another nearly straight line can be discerned.
5. Then, a somewhat less straight line from 18% to 10% can be observed from approximately 60 to 96 months of follow-up, respectively.
6. Beyond the 96-month follow-up, the line is somewhat irregular, but roughly nearly horizontal from 10% to 5% (at the 120-month follow-up).

**Figure 6:**
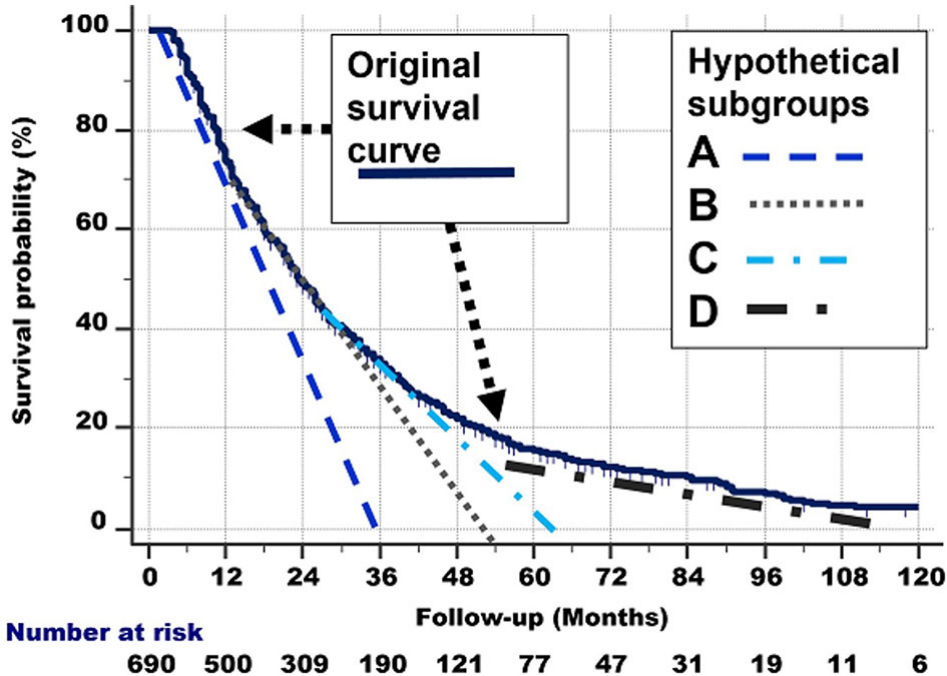
*Right:* Hypothetical delineation of the curved shape of the survival curve. See the text for details. For the sake of clarity, we have only drawn 4 tangent lines instead of 8.

The right part of **Figure 6** approximates the abovementioned graphically. For the sake of clarity, we have only drawn 4 tangent lines instead of 6.

The quantitative and graphical analyses described in the total group of 690 patients probably consists of different subgroups with different biological behaviour and survival rates.

It is important to note that these 690 individuals had the same histological type (NSCLC) and stage (IV). Thus, all had metastases at the time of diagnosis and also at the entry in this study, at least 3 months after the diagnosis. Yet, some died very quickly, (within 12 months), and others survived very long (5–10 years). The 690 “homogeneous” group in retrospect was heterogeneous, i.e., consisted of subgroups with different survival rates.

How many different subgroups exist in the 690 patients?

**Figure 7:**
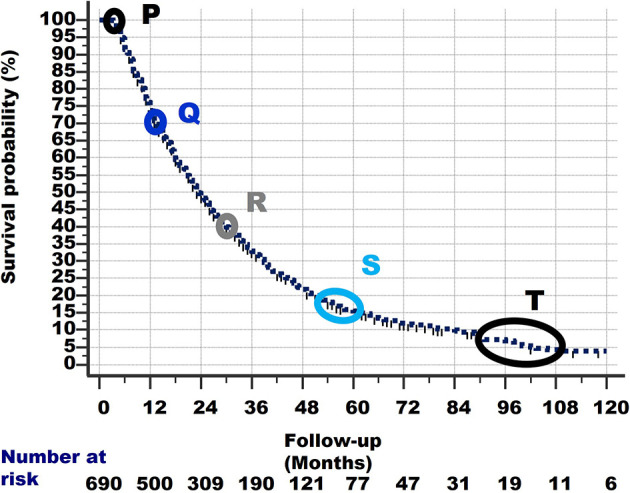
The survival curve of the 690 patients starts at Point P at 100% survival and 3 months of follow-up, as 261 other patients had already died within 3 months and are excluded from this study. From point P, the survival line shows a curved slope downward to the last point at 4% survival and 120 months of follow-up. At specific points in the survival curve, the slope shows a subtle change (i.e., becomes less steep). These points are denoted as Q, R, S and T. For details of these points, see text.

The Actual Observed survival curve gives important clues. Remember that 261 stage IV NSCLC patients from the same observation period had already died before the 3-month survival and are not considered in the current study. This explains why the survival curve of the 690 patients in **Figure 7** starts at 100%/3-month follow-up. This point is called P.

1. Closer observation shows that there are typical points in the graph, in which the slope of the survival curve shows a subtle change and becomes less steep. These points are shown in **Figure 7** and are located at:
2. Q: 70% survival/14-month follow-up,
3. R: 40%/30 months,
4. S: 18%/54–60 months
5. T: 10%-5%/90–108 months. Note that the number of patients becomes quite low after 90 months of follow-up, which could have caused the less smooth shape of the curve between 90 and 120 months of follow-up.

**Figure 8:**
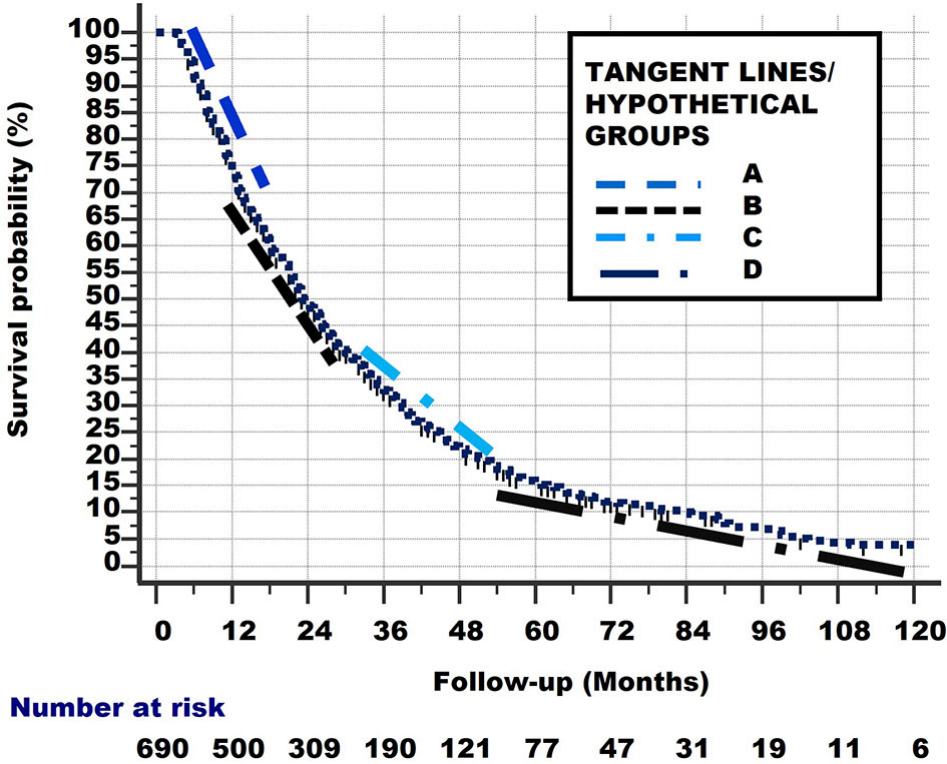
Linear lines between points P–Q, Q–R, R–S, S–T. These lines are slightly shifted up and down, and to the left and right in the figure, to make them more visible.

Linear (straight) lines can be drawn between these points (i.e., P–Q, Q–R, R–S, S–T). These are shown in **Figure 8**. These lines are slightly shifted in the figure, to make them more visible.

Of course, the subgroups which these lines represent did not start to exist at their respective starting points Q, R, S but were all present in the total group at the start of study (i.e., at point P). Consequently, the lines from **Figure 7** can be extrapolated from point P, as lines with the same slope, to the points where they cross the x-axis. These lines are shown in **Figure 8** and represent the hypothetical subgroups.

**Figure 9:**
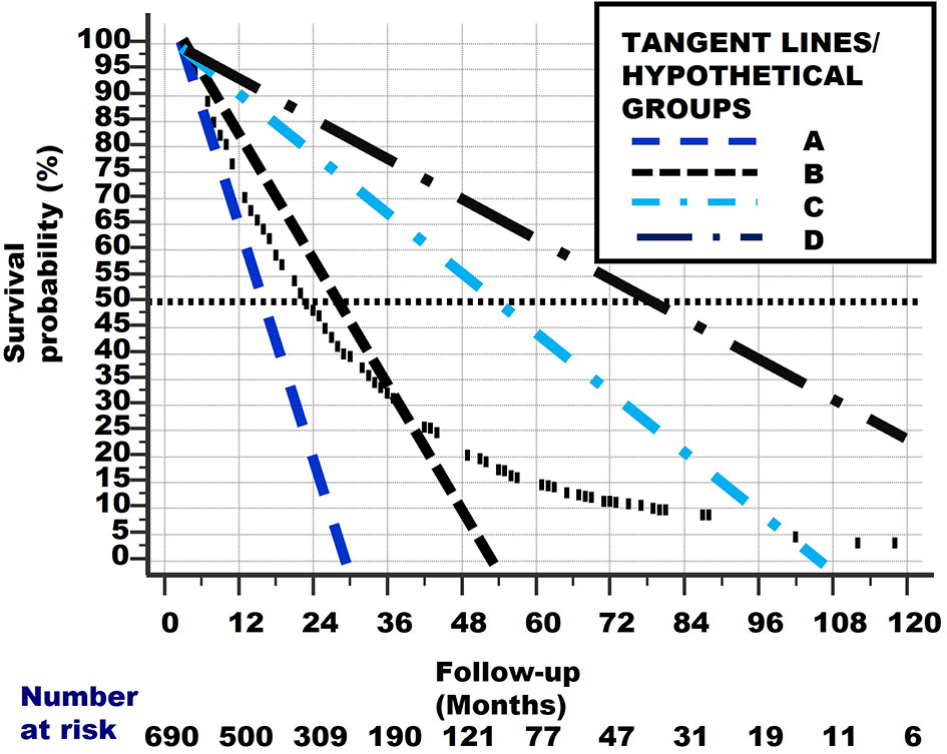
The original curved survival line is shown as small vertical black lines. The linear tangent lines of the curved survival line from **Figures 7** and **8**, between points P–Q, Q–R, R–S and S–T, are extrapolated from where they originally begun, at point P.

**Figure 10:**
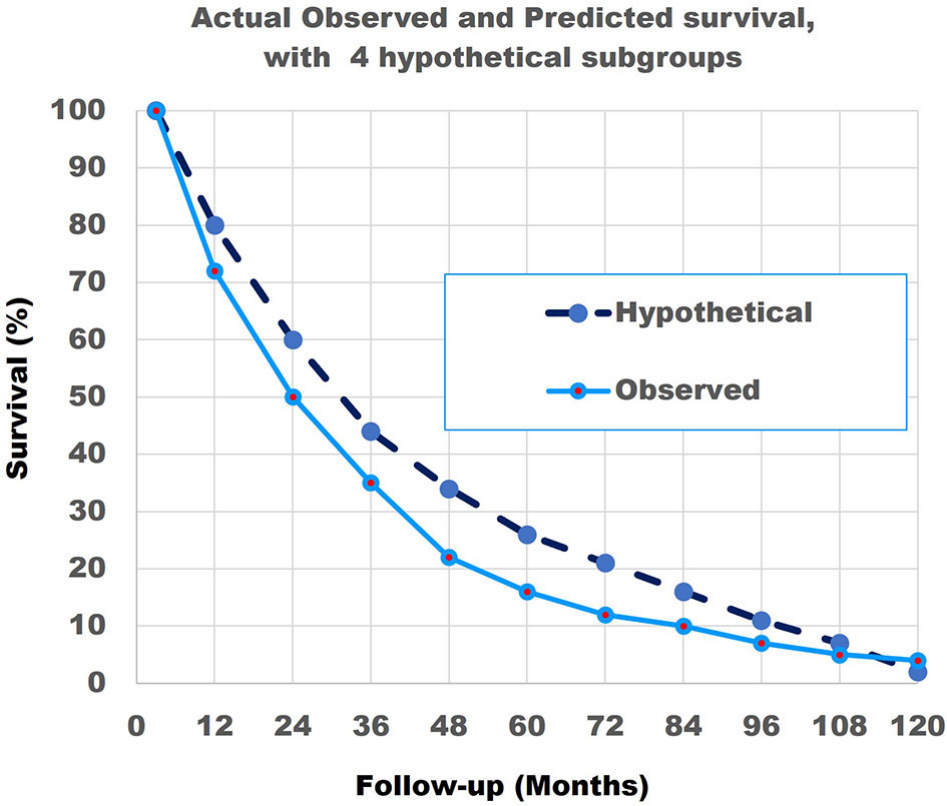
The actual observed survival curve (light blue continuous line) with the results of the combination of the model with 4 hypothetical straight lines (dark blue broken line). Note that the match is not perfect.

From **Figure 9**, we can determine the Median Survival Time and Overall Survival Time, and the percentage and total number Alive With Disease for the 4 hypothetical subgroups. **Table 1** and **Figure 10** show these data.

**Table 1:** The total number of 690 patients and the characteristics of the 4 hypothetical subgroups, derived from **Figures 7**–**9**.

Subgroup	Number	Median Survival Time (Months)	Overall Survival Time (Months)	3 Months % AWD	3 Months # AWD	12 Mo % AWD	12 Mo # AWD	24 Mo % AWD	24 Mo # AWD	36 Mo % AWD	36 Mo # AWD	48 Mo % AWD	48 Mo ¤ AWD	60 Mo % AWD	60 Mo ¤ AWD	72 Mo % AWD	72 Mo ¤ AWD	84 Mo % AWD	84 Mo ¤ AWD	96 Mo % AWD	96 Mo ¤ AWD	108 Mo % AWD	108 Mo ¤ AWD	120 Mo % AWD	120 Mo ¤ AWD
1	173	15	30	173	173	60%	104	20%	34.6	0%	0	0%	0	0%	0	0%	0	0%	0	0%	0	0%	0	0%	0
2	173	28	54	173	173	78%	135	56%	96.9	34%	58.8	12%	20.8	0%	0	0%	0	0%	0	0%	0	0%	0	0%	0
3	172	54	108	172	172	89%	153	78%	134	67%	115	55%	95	43%	74	32%	55	21%	36	8%	14	0%	0	0%	0
4	172	72	150	172	172	92%	158	84%	144	76%	131	68%	117	60%	103	52%	89	44%	76	36%	62	28%	48	10%	17
**Total AWD**	690			690	690	80%	550	60%	410	44%	305	34%	232	26%	177	21%	144	16%	112	11%	76	7%	48	3%	17
**Hypothetical % AWD**				100%		80%		60%		44%		34%		26%		21%		16%		11%		7%		2%	
**Observed % AWD in actual total group**				100%		72%		50%		35%		22%		16%		12%		10%		7%		5%		4%	

As the match of 4 hypothetical subgroups was not perfect, we then repeated the modelling study for 8 subgroups. **Table 2** shows the total number of 690 patients and their characteristics.

**Table 2:** The total 690 stage IV NSCLC patients with 3.2–120.0 months of follow-up, divided into 8 hypothetical subgroups according to the linear tangent method (see text).

Subgroup	Number	Median Survival Time (Months)	Overall Survival Time (Months)	3 Mo # AWD	12 Mo % AWD	12 Mo # AWD	24 Mo % AWD	24 Mo # AWD	36 Mo % AWD	36 Mo # AWD	48 Mo % AWD	48 Mo ¤ AWD	60 Mo % AWD	60 Mo ¤ AWD	72 Mo % AWD	72 Mo ¤ AWD	84 Mo % AWD	84 Mo ¤ AWD	96 Mo % AWD	96 Mo ¤ AWD	108 Mo % AWD	108 Mo ¤ AWD	120 Mo % AWD	120 Mo ¤ AWD
1	87	6	12	87	0%	0	0%	0	0%	0	0%	0	0%	0	0%	0	0%	0	0%	0	0%	0	0%	0
2	87	15	30	87	67%	58	33%	29	0%	0	0%	0	0%	0	0%	0	0%	0	0%	0	0%	0	0%	0
3	86	24	48	86	75%	65	50%	43	25%	22	0%	0	0%	0	0%	0	0%	0	0%	0	0%	0	0%	0
4	86	30	60	86	80%	69	60%	52	40%	34	20%	17	0%	0	0%	0	0%	0	0%	0	0%	0	0%	0
5	86	36	72	86	84%	84	67%	67	50%	50	33%	33	16%	16	0%	0	0%	0	0%	0	0%	0	0%	0
6	86	42	84	86	87%	86	74%	72	61%	58	48%	43	35%	29	17%	15	0%	0	0%	0	0%	0	0%	0
7	86	48	96	86	88%	88	76%	76	64%	64	52%	50	40%	34	28%	18	16%	9	0%	0	0%	0	0%	0
8	86	60	150	86	90%	77	80%	69	70%	60	60%	52	50%	43	40%	34	30%	26	20%	17	10%	9	5%	4
**TOTAL ALIVE**	690			690		527		407		288		195		122		67.4		34.8		17.2		15		4
**Hypothetical % AWD**				100%		70%		53%		38%		26%		18%		9%		5%		3%		2%		1%
**Observed % AWD**				100%		72%		50%		35%		22%		18%		15%		12%		8%		7%		5%

8 hypothetical subgroups are also determined by the linear tangent method used in **Figures 7** and **8**.

**Figure 11:**
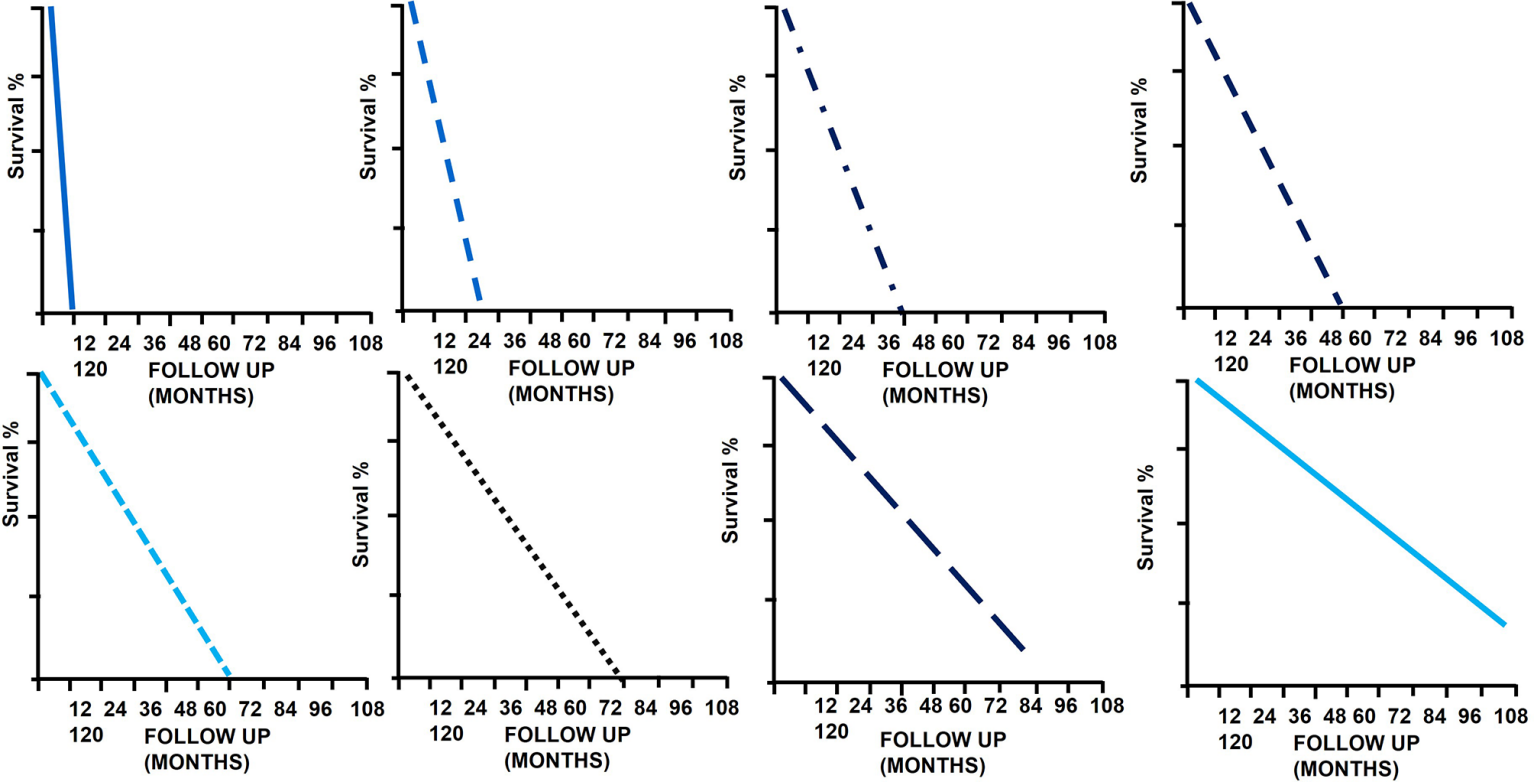
The survival curves of the 8 hypothetical subgroups.

**Figure 11** shows the survival curves of these 8 hypothetical subgroups.

**Figure 12:**
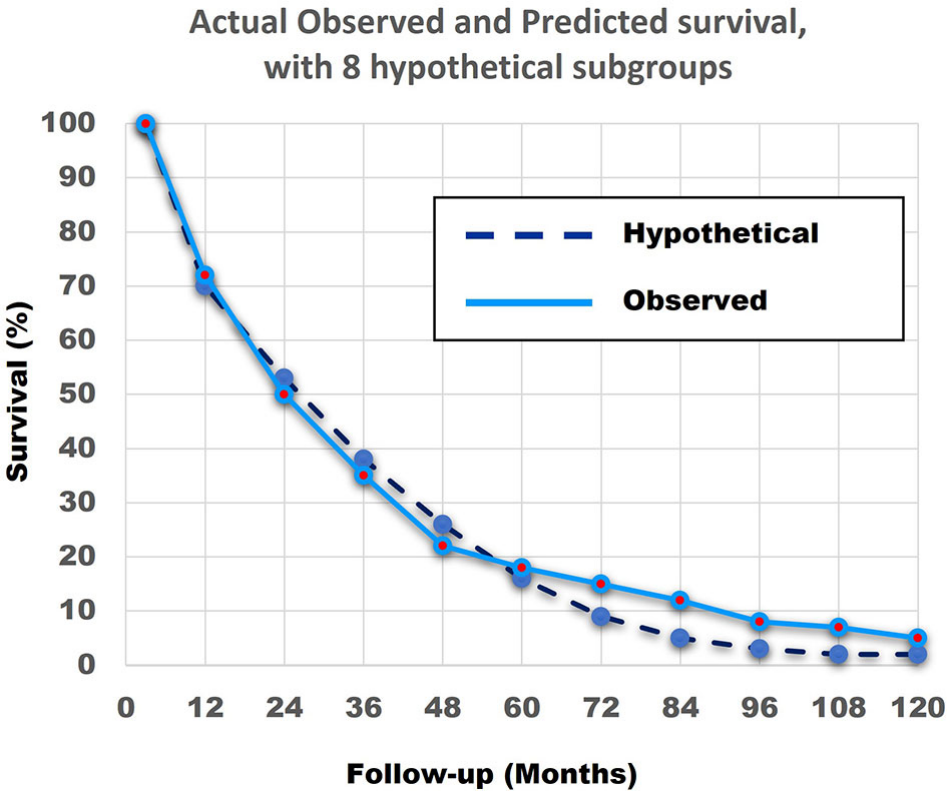
The actual observed survival curve (light blue continuous line) with the results of the combination of the model with 8 hypothetical straight lines (dark blue broken line). The similarity between the Actual Observed survival curve and the Hypothetical Calculated survival curve from the 8 hypothetical subgroups, with the linear survival curves from **Figure 11**, is remarkable.

**Figure 12** shows that the match of the theoretical line, with the original survival curve of the 690 patients, is close to perfect.

In summary, the abovementioned shows that a combination of patients with linear non-curved survival curves with different survival rates can result in a curved survival line which is very close to the Actual Observed survival curve of the 690 patients. Secondly, it is highly probable that at least 4 and more likely 8 different subgroups with very different survival rates exist in the 690 NSCLC Stage IV patients.

## Data Availability Statement

The original contributions presented in the study are included in the article. Further inquiries can be directed to the corresponding authors.

## Author Contributions

JB: concept of study and article, analysis, interpretation of results; drafting of the article and revising it critically for important intellectual content; final approval. HL: revising of the manuscript critically for important intellectual content; final approval. HG: conception and design of the study; analysis and analysis support; interpretation of data; drafting of the article or revising it critically for important intellectual content; final approval. All authors contributed to the article and approved the submitted version.

## Funding

This study was funded by a personal grant no. 2021-177 to JB from Medical Practice Dr. Jan Baak Inc., Tananger, Norway, to participate in this study and for the translation correction and publication costs.

## Conflict of Interest

The authors declare that the research was conducted in the absence of any commercial or financial relationships that could be construed as a potential conflict of interest.

## Publisher’s Note

All claims expressed in this article are solely those of the authors and do not necessarily represent those of their affiliated organizations, or those of the publisher, the editors and the reviewers. Any product that may be evaluated in this article, or claim that may be made by its manufacturer, is not guaranteed or endorsed by the publisher.
